# Ratbot navigation using deep brain stimulation in ventral posteromedial nucleus

**DOI:** 10.1080/21655979.2019.1631103

**Published:** 2019-06-16

**Authors:** Sina Khajei, Vahid Shalchyan, Mohammad Reza Daliri

**Affiliations:** Neuroscience & Neuroengineering Research Lab., Biomedical Engineering Department, School of Electrical Engineering, Iran University of Science and Technology (IUST), Tehran, Iran

**Keywords:** Ratbot, rat-robot, Deep Brain Stimulation (DBS), constant current, navigation, Ventral Posteromedial Nucleus (VPM)

## Abstract

Deep Brain Stimulation (DBS) is a medical-practical method and has been applied to solve many medical complications. Animal usage as sensors and actuators, mind-controlled machines, and animal navigation are some of the non-medical DBS applications. One of the brain areas used in ratbot navigation is the Ventral Posteromedial Nucleus (VPM), which creates non-volunteer head rotation. Rat training by water/food restriction can be used to create forward movement. In this study, a combination of VPM stimulation and water/food restriction has been employed to establish a complete navigation system. Five rats responded to VPM stimulations. However, with three of them, rats rotated to the same direction after the stimulations of either VPM side of the brain. Two rats rotated bilaterally, proportionate to the VPM stimulation side. These two rats were trained in a T-shape maze and became ratbots. The results of the 3-session test showed that their navigation performances were 96% and 86%, respectively. These ratbots are suitable for navigational purposes and are ready to complete the missions that are dangerous or impossible for humans.

## Graphical Abstract

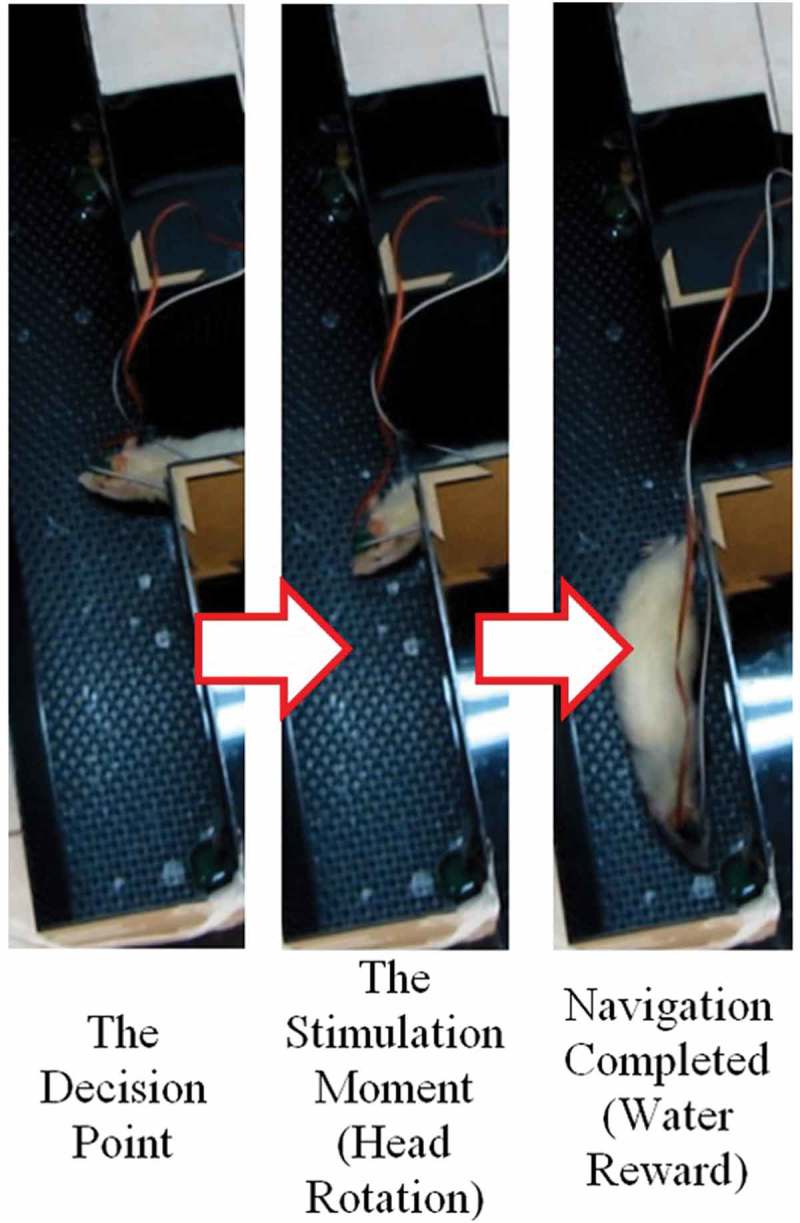

## 1. Introduction

Deep Brain Stimulation (DBS) is a medical-practical method that has been proven to have many successful applications. Some of the DBS medical applications include treatment of the Parkinson’s disease, Essential Tremor, neuropsychiatric disorders (such as Tourette syndrome, and aggressive behavior), obsessive-compulsive disorder, and obesity [1]. Some of the non-medical applications are the usage of animals as sensors and actuators, mind-controlled machines [2], and animal navigation [3,4,5]. Rat navigation has many applications in search and rescue operations in disaster areas, and explosive or landmine detections [3]. There are many advantages of using ratbots over robots in terms of mobility, perceptivity, adaptability, and energy consumption [6]. The rats that are being used for navigational purposes are called ratbots [7], rat-robots [8], or rat cyborgs [3].

Talwar et al. [9] were the first researchers who completed the rat navigation procedure. They used the Medial Forebrain Bundle (MFB) to create forward movement, as well as a reward center for correct rotations or forward movements. Moreover, the Primary Somatosensory Cortex (SI or S1BF) was used to create rotation. Other groups continued the navigation research. In 2006, a biosensor network was proposed to ease the exploration process by using rats [10]. In 2011, and then in 2013, the control commands made by human operators were analyzed to propose an automatic control model for ratbots [8,11 respectively]. In 2015, a computational model was presented to model the ratbot locomotion based on the cyborg intelligence [7]. In 2016, a maze solving comparison among the normal rats, the ratbots and the computer was implemented. The comparison factors were the steps, the spent time (comparison only between normal rats and ratbots), and the coverage rates. The results showed that the ratbots were superior in maze solving than the others [6]. In 2017, ratbot rotations were controlled by the human controller’s steady-state visually evoked potentials (SSVEPs). The brain-to-brain interface (BBI) system used in this study was inclusive of an electroencephalography (EEG) device, a server, a brain stimulator, and a graphical user interface (GUI). The server analyzed the EEG signals to figure out the participants’ preferred direction, i.e. the left or right path selection. Then, the electrical stimulation was applied to the electrodes accordingly. The 82.2% communication performance was promising for the untrained animals controlled by humans [12].

In addition to MFB and SI, used by Talwar et al. [9], other brain areas were used to complete the navigation. In a study, the Amygdala and the Ventral Posterolateral Nucleus were used for the navigation of rats. The Amygdala was employed to create forward locomotion and the Ventral Posterolateral Nucleus (VPL) was used to produce rotation [13]. The Periaqueductal Gray (PAG) is another area that is proposed for forward locomotion [14]. However, in a study, the Dorsolateral Periaqueductal Gray (dlPAG) was used as the stop command. Increasing the stimulation intensity produced alertness, freezing, and finally escaping behavior [15]. Contrary to research [15], study by Wang et al. [14] used the area to create forward movement. This study employed the Intercollicular Nucleus (ICo) of the pigeons. This area in avian is thought to correspond to the PAG in mammals [14]. Therefore, the PAG should be considered as a potential area for forward movement in rats.

The Ventral Posteromedial Nucleus (VPM) is another brain area that is used for rotational purposes. Creating virtual touch in order to initiate non-volunteer rotation is the main goal of the investigation [16]. In this study, the VPM stimulation performance is compared to the MFB and SI stimulation. The results show that there is a thorough superiority in using VPM for rotational purposes. The post-training performance of the left rotation for the SI stimulation was 81% and the right rotation performance was 91%, while the VPM stimulation performance could reach the 100% rotation performance without any training. The reason is that the VPM stimulation causes a larger SI area activation, in relation to the direct SI stimulation. It should be noted that the SI rats need to retrain every week to keep their performance, but the VPM rotation performance is relatively stable. The VPM stimulation amplitude and the total stimulation time have significant effects on the rotation angle. The increase in their values enhances the rotation angle linearly [16].

Some brain areas can be used as clues for locomotion. Rats do not necessarily move forward after receiving an MFB stimulation; e.g. they may press a bar in an operant chamber to receive an MFB reward without moving forward. After several days of training and building a connection between post-stimulation forward movements and receiving rewards, the rats learn that the MFB stimulation is related to the forward movement. Then, right after stimulations, the rats move forward voluntarily to receive a reward [17,18]. This is called volunteer locomotion. In contrast, stimulation of some brain areas, such as VPM, VPL, Amygdala, and dlPAG, creates non-volunteer locomotion; therefore, no training is needed for the movements. The rats move or rotate instantly due to the stimulations. When volunteer locomotion is intended, rat training is necessary. The rotation performance results of SI stimulation, for example, become better when a rat is trained to obtain a reward after a correct turn [16]. In addition, rats can be trained to follow the intended orders by water/food restriction. Rats learn the water-restricted mazes faster than the food-restricted mazes [19]. The water restriction training can be carried out by removing the water for a day (this needs a careful monitoring) or by giving unpalatable water to the rat. Adding 1–5% of citric acid to the water can nearly bring the same training performance as the water restricting without producing any signs of dehydration or serious health problems even after six months of the unpalatable water consumption. The rats consume about half of their normal consumption level and are ready to receive plain water as a task reward [20].

The initial focus on navigation has been on the combination of the MFB and SI in most studies. The drawbacks of SI such as the non-reliability caused by the non-100-percentage correct rotation performance as well as the need for continuous retraining have led to newer studies on the VPM investigation. VPM has solved the addressed problems; however, there are other problems associating with the combination of VPM and MFB. The coordinates of the VPM electrode entrance hole are very close to the MFB. Therefore, the usage of two separate connectors is bound to be limited. Mounting two pairs of electrodes on one connector is also impractical due to the variable bregma-lambda distance in different rats; consequently, manufacturing prefabricated connectors creates significant placement errors. Moreover, manufacturing a connector during the surgery after the bregma-lambda distance measurement is also impractical because of the short surgery duration. Due to the addressed problems, simultaneous utilization of VPM and MFB seems impractical and an MFB substitutional brain area or an external motivation should be employed to create forward locomotion. In addition, VPM can produce rotations and cannot be used alone to navigate rats. Accordingly, to complete the previous researches in our study, we have combined the VPM stimulation and water/food restriction to accomplish the navigation.

## 2. Material & methods

### 2.1. Animals

Five wistar rats were used in this study. The Weights of the rats were between 280 and 370 grams (328 ± 29). The wistar rats were selected in order to reduce the visual direction selection bias. In addition, male rats were selected because of their bigger brains so that the sizes of the brains would be better matched with the rat brain atlas prepared by Paxinos and Charles [21]; and the surgeries and the electrode placements were implemented more conveniently. The Institutional Animal Care and Use Committees (IACUCs) laws have been applied in the study.

### 2.2. Electrodes and devices

**Figure 1.**
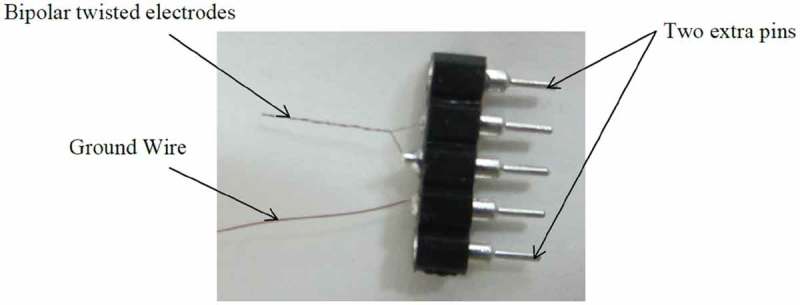
Bipolar-twisted electrodes. Two extra pins were considered in order to prevent the electrode and the ground wire pins to get damaged by the rats.

Two bipolar-twisted stimulation electrodes were fabricated manually from coated nichrome wires (A-M Systems, USA) for each rat (Figure 1). The coated wire diameter was 0.0026 inches, and the bare diameter was 0.002 inches. The connectors had five pins, two for the bipolar electrodes, one for the ground connection, and two extra pins in order to prevent the rat nail damages to the pins of the electrodes and the ground wire. The stimulation device (Blackrock Microsystems, USA) had the output voltages between 4.7 and 9.5 V, the electric currents between 1 and 215 microamperes, and the frequency of 4 Hz to 5 kHz. This device automatically measured the impedances of the electrodes whenever needed.

### 2.3. Surgery procedure

The first two rats were anesthetized using 25 mg/kg Xylazine, and 100 mg/kg Ketamine. The injections were made through the Intraperitoneal (IP) area. Both of them gained some levels of consciousness during the surgery; therefore, 50 mg/kg of Ketamine redose was needed. Other rats were anesthetized with 50 mg/kg of Xylazine, 50 mg/kg of Meloxivet, and 400 mg/kg of Ketamine. The substances were injected into the thigh muscle. This anesthetization process led to a deeper anesthetization and redosing was not needed anymore. After the surgery, 500 mg/kg of dextrose saline and 50 mg/kg of Enrofloxacin were injected into the rats’ IP.

**Figure 2.**
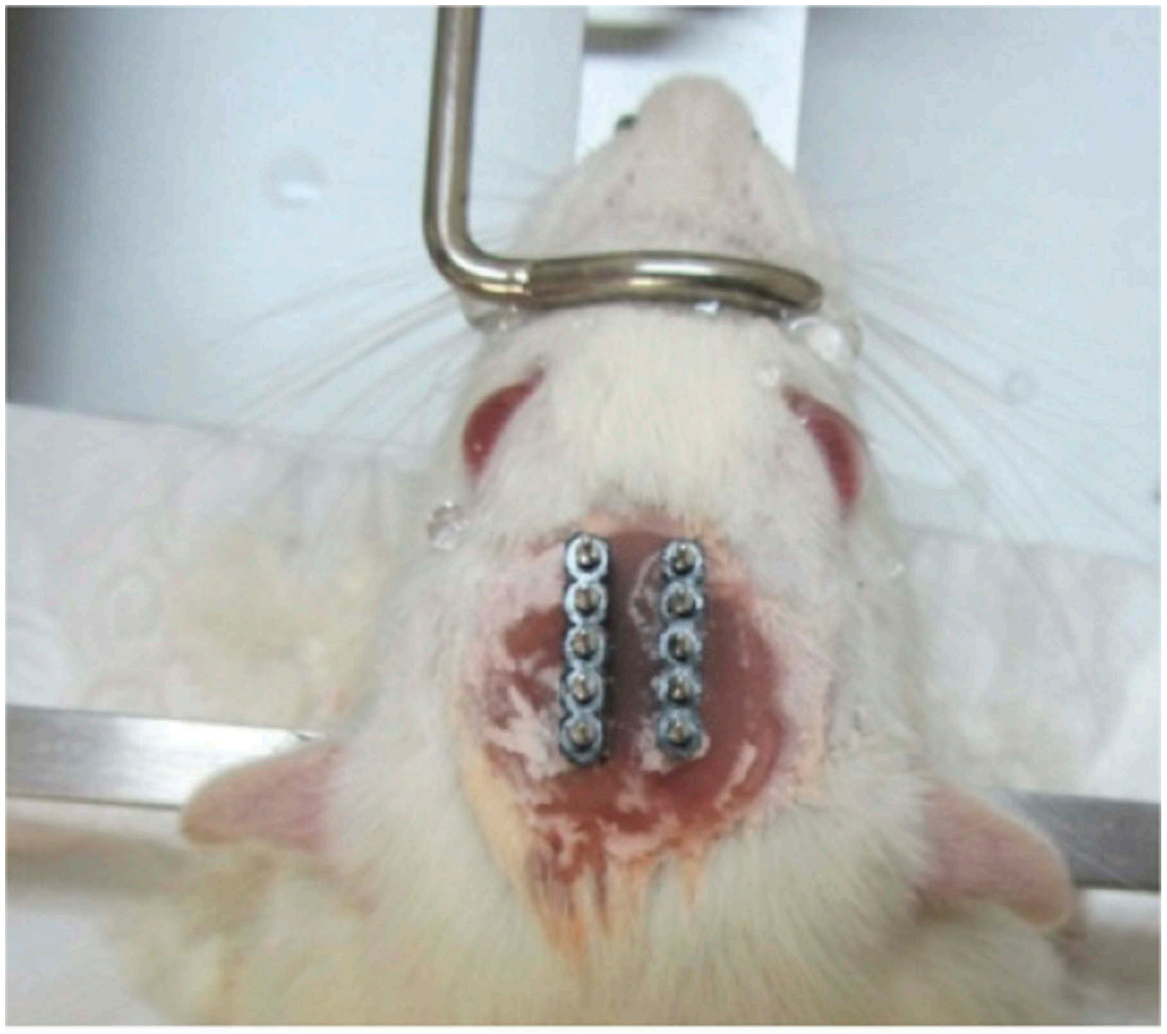
A rat at the final surgery stage. Two VPM electrodes are placed in the head.

**Figure 3.**
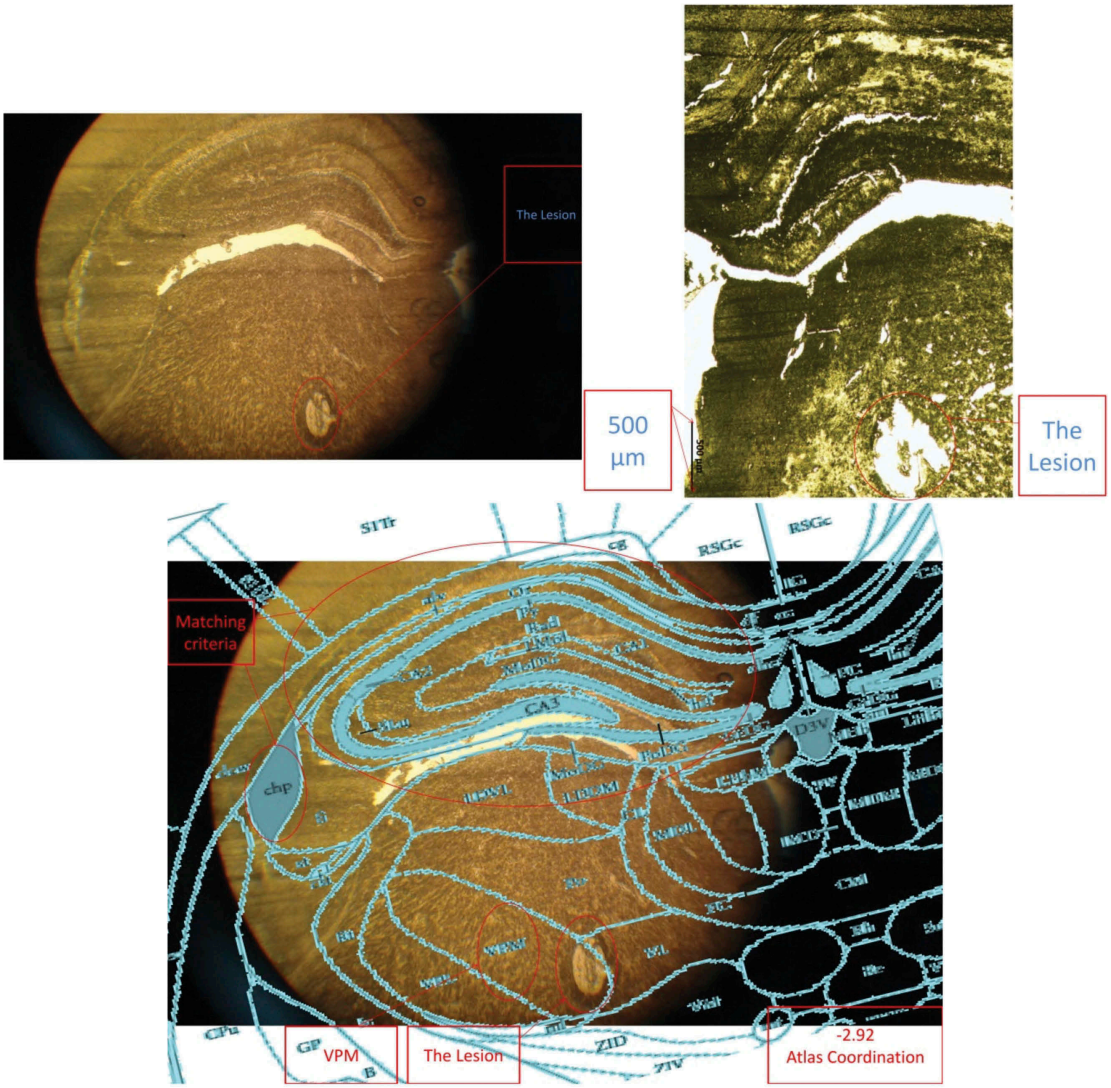
A histology image example (placement confirmation of the left side electrodes).

After fixing the head in the stereotaxic apparatus, an incision was made at the midline of the scalp. Then, the tissues over the skull were removed by H_2_O_2_ and the skull was cleared by ethanol, and water. Then, two craniotomies for the electrodes, and four to six craniotomies for the stainless steel screws, as anchorages, were drilled. The electrodes were placed in the bilateral VPM areas (AP: −2.8; ML: ± 2.6; DV: 6) and the dental acrylic was applied to fix the electrodes (see Figure 2). The electrode placements were approved behaviorally and histologically (see Figure 3). Rats had at least 7 days of post-surgery recovery.

### 2.4. Stimulation parameters

After the recovery, a pulse train containing 20 or 25 pulses with the amplitude of 50 µA, the pulse width of 100 ms, the pulse interphase of 120 ms, and the frequency of 100 Hz was used as the starting stimulation train. In case a rat did not respond to the stimulation, the amplitude was raised by 50 µA, or the pulse width was raised by 50 ms. Since our device could not produce pulses with amplitudes higher than 215 µA, the highest amplitude was kept at the 200-µA value and the further increments were applied by the pulse width raises. The rats that did not show any rotation with different stimulation values were excluded from the study. When a rat responded to a stimulation, the amplitude, the pulse width, the pulse interphase, and the frequency were kept constant and the number of pulses was adjusted to control the rotation angle. In other words, the rats should have rotated enough to be directed to the correct path. Therefore, 20 to 200 pulses were used as the number of pulses for different rats or in different sessions. The impedances of the electrodes and the used parameters were recorded every session by the stimulation device for further analysis (see Tables 1 and 2).

**Table 1.** The Stimulation Parameters of the First Ratbot.

	Day 1^a^	Day 9^b^	Day 42^c^	Day 46	Day 47	Day 49	Day 50^d^	Average
Left electrodes impedance (kΩ)	66 & 76	83 & 87	52 & 66	68 & 80	62 & 72	84 & 91	93 & 97	73 & 81
Right electrodes impedance (kΩ)	38 & 40	45 & 72	31 & 50	19 & 26	43 & 58	47 & 66	69 & 73	42 & 55
Left side number of pulses	-	100	50	25	25	25	25	42
Right side number of pulses	-	200	100	70	50	20	20	77
Left side Amplitude (µA)	-	100	100	100	100	100	100	100
Right side amplitude (µA)	-	100	100	200	200	200	150	158
Left side pulse width (ms)	-	200	100	200	200	200	100	167
Right side pulse width (ms)	-	200	300	500	300	250	200	292
Left side interphase (ms)	-	120	120	120	120	120	120	120
Right side interphase (ms)	-	120	120	120	120	120	120	120
Left side frequency (Hz)	-	100	100	100	100	100	100	100
Right side frequency (Hz)	-	100	100	100	100	100	100	100

The Stimulation Parameters were obtained each day according to the instructions explained in Section 2.4.. We changed the parameters every session to obtain the suitable rotational behavior. ^a^Surgery date; ^b^after at least a week of recovery; ^c^first training session; ^d^last test session.

**Table 2.** The Stimulation Parameters of the Second Ratbot.

	Day 1^a^	Day 7	Day 10^b^	Day 31^c^	Day 32	Day 33	Day 34^d^	Average
Left Electrodes Impedance (kΩ)	72 & 81	90 & 94	168 & 173	79 & 80	48 & 65	80 & 83	70 & 77	87 & 93
Right Electrodes Impedance (kΩ)	75 & 84	97 & 106	95 & 169	68 & 80	83 & 93	73 & 79	72 & 80	80 & 99
Left side number of pulses	-	100	100	70	70	70	70	80
Right side number of pulses	-	200	200	50	70	70	70	110
Left Side Amplitude (µA)	-	200	200	100	100	100	100	133
Right Side Amplitude (µA)	-	150	150	200	200	200	200	183
Left Side Pulse Width (ms)	-	200	200	100	200	100	100	150
Right Side Pulse Width (ms)	-	300	300	400	300	300	300	317
Left side Interphase (ms)	-	120	120	100	100	100	100	107
Right side Interphase (ms)	-	100	100	100	100	100	100	100
Left Side Frequency (Hz)	-	100	100	100	100	100	100	100
Right Side Frequency (Hz)	-	150	150	100	100	100	100	117

The Stimulation Parameters were obtained each day according to the instructions explained in Section 2.4. We changed the parameters every session to obtain the suitable rotational behavior. ^a^Surgery date; ^b^after at least a week of recovery; ^c^first training session; ^d^last test session.

### 2.5. Maze and training and test procedures

To train the rats and to test their navigation performance, a T-shape maze was used. Water or food rewards were put at the end of the maze corners.

The suitable stimulation parameters were obtained for each rat in the process explained in the 2.4. section. The next phase was the restriction phase. The rats were restricted from food or received unpalatable water (adding 5% of citric acid to water, as the water restriction method) to be prepared for the training sessions. Then, to reduce the anxiety of the rats, they were put into the maze to roam freely to adapt with the maze environment (1 to 3 sessions). Water/food was available at both ends of the T-shape maze arms and the rats learned that they could resolve their needs by finishing the maze. In the training phase (1 to 3 sessions, depending on the learning speed), the electrodes of the rats were connected to the stimulation device via a cable. The water-/food-restricted rats were put at the starting point of the maze and they searched for their needs. At the decision point (intersection of the T-shape arms), the rats were stimulated in one of their VPM areas to select our desired direction. These directions were randomly chosen in order to minimize the fortuitous path selection. In case the rats chose to continue to the desired path, the rewards were available for them for seconds. Then, they were put at the starting point once again for a new trial. Sometimes, stimulations rotated rats’ heads, but they did not continue to the desired path and turned their heads voluntarily toward the wrong direction. In this case, the second VPM stimulations were sent to their brains to make them rotate toward the correct direction once again. Tandem stimulation, however, was not possible because of the neural refractory period. This multi stimulating method was effective in some trials. In a few trials, even after receiving multi stimulations, the rats finally chose to continue to the wrong path. In these cases, they were derived from the rewards and were put at the starting point to start a new trial. These training sessions continued until the rats realized the correlation between electrical stimulations and the respective paths. Ultimately, three test sessions were conducted to compute their navigation performances. The navigation performance is the number of correct decisions, proportionate to the total trials.

## 3. Results

### 3.1. Stimulation parameters and exclusion criteria

Five rats were used in this study. Three rats showed the same head rotation as a result of the stimulation applied to each of their brain VPMs. They were approvable in terms of rotation, but they were not suitable for navigation. Therefore, they were excluded from the rest of the process, i.e. they were not put into the maze for navigation performance computation. Two rats showed the desired rotations. The suitable stimulation parameters were obtained every training or test session, according to the method explained in the 2.4. section. The parameters changed every session despite using constant current stimulation method. The obtained values are shown in Table 1.

**Figure 4.**
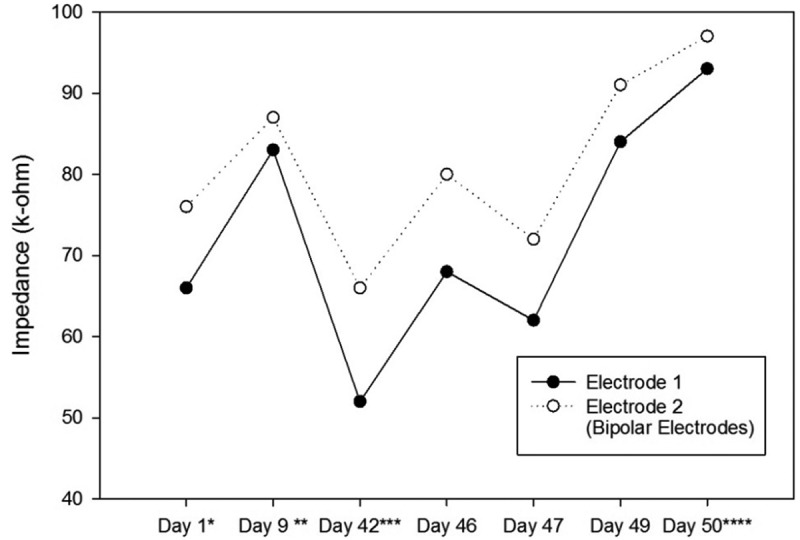
Impedance Changes over Time for the Left Electrodes of the First Ratbot. There is a 79 & 47% impedance increment during the 9 days of training and testing. * Surgery date; ** after at least a week of recovery; *** first training session; **** last test session.

**Figure 5.**
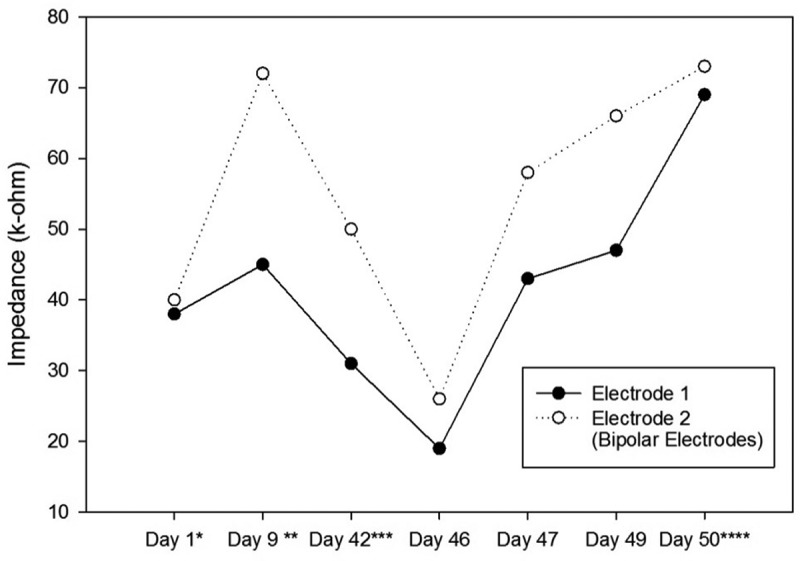
Impedance Changes over Time for the Right Electrodes of the First Ratbot. There is a 123 & 46% impedance increment during the 9 days of training and testing. * Surgery date; ** after at least a week of recovery; *** first training session; **** last test session.

**Figure 6.**
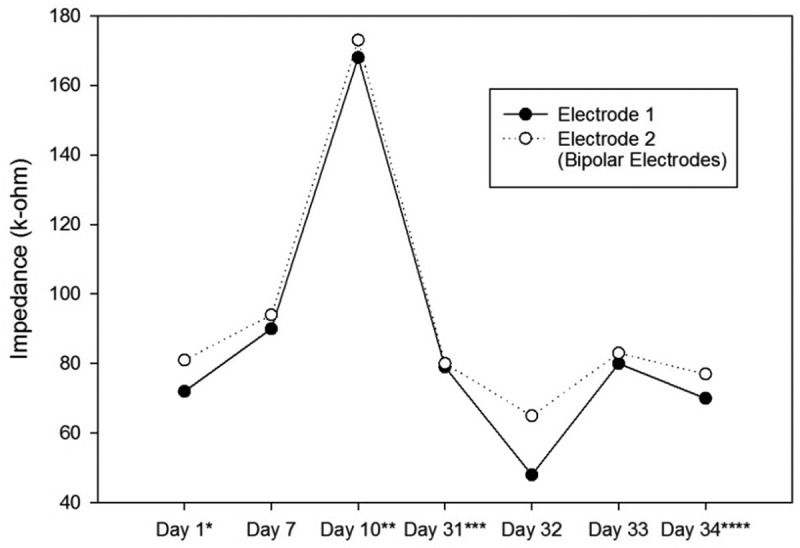
Impedance Changes over Time for the Left Electrodes of the Second Ratbot. The impedance of the electrodes remained relatively constant over the 4 days of training and testing (11 & 4% decrease). * Surgery date; ** after at least a week of recovery; *** first training session; **** last test session.

**Figure 7.**
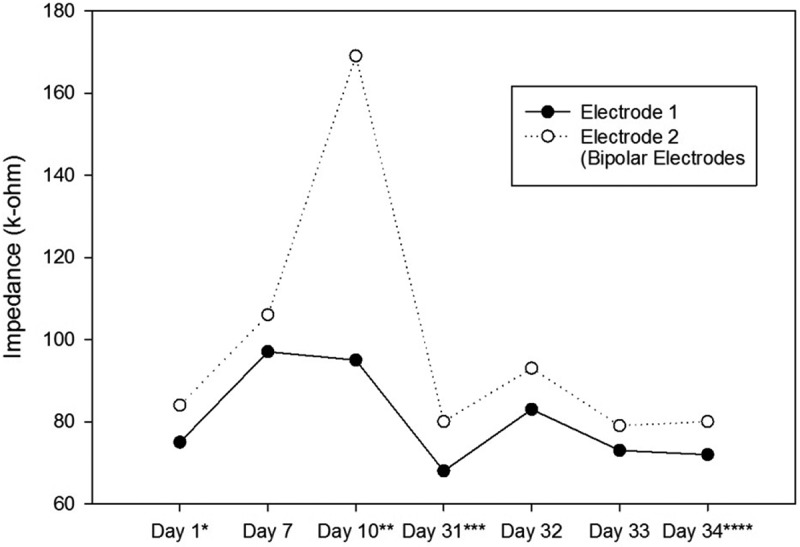
Impedance Changes over Time for the Right Electrodes of the Second Ratbot. The impedance of the electrodes remained relatively constant over the 4 days of training and testing (6 & 0% decrease). * Surgery date; ** after at least a week of recovery; *** first training session; **** last test session.

Impedances of the electrodes are expected to rise over time. There is a significant impedance increment of the electrodes two weeks after the surgery, caused by tissue reactions. Then, there is a rapid decrease, followed by fluctuating increment and decrement trends over 100 days after the surgery. These changes are the results of electrode and tissue components of the overall impedance [22]. Figure 4–7 show the impedance changes of the electrodes over time. The impedances of the first ratbot electrodes showed significant increments in the 9 days of training and test sessions. The impedances of the second ratbot electrodes, however, were relatively constant in the 4 days of training and test sessions.

### 3.2. Navigation performance

The navigation performances of the two rats were calculated based on the number of correct rotations proportionate to the total number of trials. The results are shown in Tables 3 and 4. According to the results, the first ratbot could find the correct path with the performance of 96% and the second ratbot with the performance of 86%.

**Table 3.** The Navigation Performance of the First Ratbot.

	Test Session		
	#1	#2	#3
Correct Rotations	13	25	10
Incorrect Rotations	1	0	1
Performance (%)	93	100	91
Total Performance (%)	96

**Table 4.** The Navigation Performance of the Second Ratbot.

	Test Session		
	#1	#2	#3
Correct Rotations	13	14	16
Incorrect Rotations	3	1	3
Performance (%)	81	93	84
Total Performance (%)	86		

## 4. Discussion

Two rats completed the navigational tasks to become ratbots. The results showed that the VPM of the brain can be employed to obtain high navigational performance. Combining the VPM high performance with the water/food reward enables us to make a complete rat navigation system.

Our stimulation device produces current stimulations. These are suitable stimulations because the total charge is constant, regardless of the impedances of the electrodes. We used bipolar electrodes and biphasic stimulation to reduce any tissue damage. The biphasic stimulation can be continuously applied to the tissue for 36 h without any damage if the charge per phase is below 0.45 µC [23]. Considering that our strongest stimulation consists of 500-µs width and 200-µA amplitude per phase pulses, our maximum charge per phase will be 0.1 µC. Therefore, our charge per phase is below the threshold and the stimulation can be applied to the brain continuously without any damage. In should be noted that when microelectrodes are used, the damage is only estimated by the charge per phase factor, and not the charge density or other factors [24].

Tissue reactions cause a significant impedance increment of the electrodes two weeks after the surgery. Then, following a rapid decrease, fluctuating increment and decrement trends remain for at least 100 days after the surgery [22]. In an agreement with this study, we observed a significant increase in our electrode impedances after one week following the surgeries in both rats. Then, the impedance levels were turned back to the normal. Furthermore, the impedances of the electrodes of the first rat increased in the long period (Figures 4 and 5). In contrast, the impedances of the electrodes of the second rat remained constant in the long period (Figures 6 and 7). Our observations confirm the fluctuation phenomenon.

The constant current stimulation method is preferred to constant voltage stimulation method because the former counteracts the clinical effects caused by impedance changes over time [25]. Constant current stimulation keeps the volume of tissue activated (VTA) constant. While clinical outcome superiority of the constant current stimulation method is not quite proven [26,27], many companies prefer to build constant current stimulators because of the safety assurances [26]. Some studies have reported changes of their stimulation settings despite using constant current stimulation method. One of the patients (10% of the total patients) of study [28] has requested changes to his initial stimulation parameters three months after the surgery. In addition, one study reports mild changes in verbal fluency and processing speed over time in the Parkinson’s Disease patients who were treated by using Subthalamic Nucleus (STN) DBS. The changes were only visible in the stimulation group and the effects were attributed to both electrode placement and stimulation [29]. These findings may suggest that the physiological effects due to stimulations may change over time despite using constant current stimulation method. Consistent with these studies, we had to change our stimulation parameters every session in order to see suitable rotational behavior. However, the constant current stimulation method outcomes and the reasons behind the need for parameter changes over time should be investigated in controlled pilot studies in the future.

Our stimulation parameters are higher than those applied to VPL area in study [13] in terms of pulse numbers, but the rest of the parameters are similar. Our parameters are higher than the applied values to stimulate the MFB and SI areas and in studies [18,30], in terms of pulse numbers, they are lower in terms of the pulse width respectively, while the rest of them are similar. Our pulse numbers and width choices state that we have used similar stimulation time as in study [16] that has stimulated VPM. The minimum stimulation time to start the rotation in the latter study is similar to ours.

Stimulating VPM caused immediate rotation in our study. In other words, in 100% of our stimulations, the rats rotated. This result is in agreement with study [16]. This is the main reason to substitute the SI area with the VPM to have higher and steadier navigation performance. The SI group (BF group) in study [16] had a maximum pre-training rotation performance of 60% (34% average); and 90% at most (81% average) after the training. Other studies have not reported out of the maze rotation performances. The T-shape maze solving performances are our main results. The performance of the first ratbot was 96% and it was 86% for the second ratbot. Wang et al. [17] has reported the SI rotation performances of 86% and 89% (86 & 89 successful turns in the 100 total turns) in the automatic control for their two rats in the maze. In addition, manual control had the performances of 95% and 92% in the same rats. In addition, study [8] has applied SI to create rotations in six rats and has reported the navigation performances between 77.8% and 100% (90% average) in human controlling and 67.2–100% (83% average) in automatic controlling. These results show that although the SI performances are lower than VPM performances in out of maze rotations, they can increase in mazes. We suggest that the rats’ environmental perception is effective in SI rotations. In other words, SI acts as a cue. When rats are stimulated out of mazes, they sometimes choose not to rotate voluntarily. Nevertheless, when they are in mazes, they interpret SI stimulations as boundaries or barriers; consequently, they rotate. Using VPM, however, has the advantage of having steady rotation performance in and out of mazes.

The VPM stimulation creates non-volunteer rotations. When the rats reach the T-shape-maze decision point, they receive a single stimulation. This causes their head to rotate toward the correct path. However, the rats sometimes turn their heads voluntarily toward the wrong direction, despite trainings to accompany the stimulations and the decisions. Therefore, it is needed to stimulate their brain once more. In rare cases, the brains need to be stimulated multi-times to rotate toward the right direction. Finally, when they choose to continue the correct path, they do not receive additional stimulations. This problem is the main limitation of non-volunteer navigation. In other words, contradictions between volunteer and non-volunteer decision-makings create difficulties in navigation.

To the best of our knowledge, there is only one valid previous study studying VPM for navigational reasons [16]. There are differences between our results and this study. In the study by Xu et al. [16], it is stated that SI stimulation causes ipsilateral turning behavior, contralateral turning behavior or no obvious response but all the VPM stimulations have created ipsilateral rotations. In contrast, we had three rats which had an ipsilateral rotation by one side stimulation, and contralateral rotation as the result of the other side stimulation. This phenomenon caused three of our rats to perform a one-sided rotation as the result of both side stimulations. This can be considered as another limitation of using VPM for navigational purposes. Swenson [31] suggests that touch and pressure touch, pressure and vibration sensory fibers are connected to both VPM sides. Consequently, lesion in one of these areas does not annihilate all of the face sensations of one side. This fact explains the phenomena. The reason that study [16] did not confront this problem was perhaps due to their electrode differences with ours. Their electrodes have more exposed tip surface that stimulated a larger area in VPM.

The coordinates employed by Huai et al. [13] to simulate the VPL area are actually an area inside the VPM area according to the rat brain atlas prepared by Paxinos and Charles [21]. Therefore, it seems that the study [13] has navigated the rats by their VPM instead of the VPL. Consequently, studying the VPL effect on navigation can be implemented in the future more precisely.

Kant et al. [19] has stated that the rats learn faster when they are restricted from water than food. In our study, however, we observed that the second ratbot did not ever learn the task with water restriction. This could be the result of the 5% citric acid unpalatable water restriction program proposed by study [20] that has failed on this rat or it is a contradiction to the results of study [19]. To answer this question, further investigations should be implemented.

## 5. Conclusions

The ultimate purpose of the navigation studies is to conduct complicated missions that are dangerous or impossible for humans. A combination of VPM stimulation and water/food restriction was employed to establish a complete navigation system in our study. The results of the tests in the T-shape maze showed that the navigation performances of the two ratbots were 96% and 86%, respectively. Water/food restriction and T-shape maze training were used to create forward movements, but the rotations happened non-voluntarily due to the stimulations. The impedances of the electrodes and the stimulation parameters were recorded every session to be compared with the previous studies. Other stimulation combinations or other areas can be used in future studies to improve the performance and the applicability of the combination used in this study.

## Research Highlights

- The ratbots were navigated using VPM stimulation and water/food restriction.
- A combination of DBS & behavioral methods was employed to complete the navigation.
- VPM is superior to SI for resolving the need for training, i.e. (auto-rotation).
- The VPM stimulation may create contralateral or ipsilateral turning behaviors.
- The rotational behavior changed over time despite using constant current stimulation.
